# Influence of a patient information program on adherence and persistence with an aromatase inhibitor in breast cancer treatment - the COMPAS study

**DOI:** 10.1186/1471-2407-13-407

**Published:** 2013-09-04

**Authors:** Volker Ziller, Ioannis Kyvernitakis, Dana Knöll, Astrid Storch, Olaf Hars, Peyman Hadji

**Affiliations:** 1 Department of Gynecology, University hospital of Giessen and Marburg GmbH, Marburg, Baldingerstrasse, 35033 Marburg, Germany; 2 Department of Endocrinology, Reproductive Medicine and Osteoporosis, University hospital of Giessen and Marburg GmbH, Marburg, Baldingerstrasse, 35033 Marburg, Germany; 3 Olaf Hars Statistical Consulting, Berlin, Germany

**Keywords:** Adherence, Compliance, Aromatase inhibitors, Breast cancer, Endocrine treatment

## Abstract

**Background:**

It is known that suboptimal adherence rates may affect endocrine treatments for breast cancer, but little information has been reported whether any efforts to improve treatment adherence have been successful. We designed a randomized, controlled study to investigate the effect of oral or written patient information program on adherence and persistence when receiving an aromatase inhibitor (AI).

**Methods:**

The study cohort included 181 female patients receiving an adjuvant AI treatment randomly assigned to one of three groups. The first group received reminder letters and information booklets, the second group was reminded and informed through telephone calls and the control group received neither. The primary endpoint was the rate at which patients were classified as adhering to treatment after twelve months.

**Results:**

Baseline results showed a well-balanced randomization with no significant differences between groups. After 12 months, 48% (CI 35–62) of the control group, 62.7% (CI 49–75) in the telephone group and 64.7% (CI 51–77) in the letter group were adhering to therapy. A post hoc pooled analysis with a one-way hypothesis for both interventions versus control indicated a significant difference between the groups favouring the intervention (p = 0.039).

**Conclusion:**

The aim of this study was to investigate the efficacy of a simple and practical interventional program in enhancing adherence to breast cancer treatment. Patients receiving additional/supplemental information appeared to have an improved adherence rate even though the differences between groups were not statistically significant for the primary endpoint.

## Background

In past decades, breast cancer survival rates have increased substantially as a consequence of significant improvements in early diagnosis and the introduction of more effective treatments e.g. adjuvant endocrine therapies. Third-generation aromatase inhibitors (AIs) such as anastrozole (1 mg/d), letrozole (2.5 mg/d), and exemestane (25 mg/d), have proven more effective than tamoxifen in upfront, switch and extended adjuvant treatments with regard to disease-free survival (DFS) and distant metastasis (DM) in postmenopausal women with hormone-sensitive early breast cancer [1-3]. However, patients only attain maximum benefits from their medications if they follow the instructions and adhere to dosing schedules [4]. As with other chronic diseases, patients with breast cancer often fail to take the correct dosage at the prescribed frequency and for the proper duration [5].

Awareness of poor patient adherence to treatment programs has led to investigation of this issue by an increasing number of clinical studies [6-9]. Due to the seriousness of their disease, cancer patients are (generally) considered highly motivated and compliant, but limited prospective data is available on their adherence to adjuvant treatment for breast cancer, and more specifically, regarding the use of aromatase inhibitors. Recent studies reported that up to 50% of patients stop taking their medication during the course of 5 year adjuvant treatment with tamoxifen, resulting in a significant increase in mortality [10]. It was also reported that a substantial proportion of patients on anastrozole fail to adhere to the recommended treatment after the first year. In a different report, only 49% of patients with breast cancer (BC) took adjuvant hormonal therapy for the full duration on the optimal schedule [11-16]. However, these studies were health care data based analyses and do not provide evidence about daily clinical routine treatment at the patients level [13]. We recently reported the results of a study using a combination of self-reporting and prescription refill counts in breast cancer patients taking daily tamoxifen or anastrozole. After 12 months of treatment, 80% of women on tamoxifen and 69% of women on anastrozole were adhering to treatment [17].

With a growing number of patients surviving breast cancer, the problem of adherence with therapy is becoming increasingly important [13,18]. Increased adherence and persistence are likely to improve patient outcomes, and COMPAS (Compliance in Adjuvant treatment of primary breast cancer Study) was designed to investigate the effects of frequent reminders that informed and motivated patients with respect to breast cancer and its treatment with an aromatase inhibitor. The aim of this study was to investigate the efficacy of a simple and practical intervention program on the ability of patients to stay on treatment with an aromatase inhibitor for the endocrine treatment of primary breast cancer.

## Methods

### Objectives and definitions

In the absence of a reliable treatment-specific maker, measuring patient adherence and persistence is a complex challenge that (in a real life setting) can only be achieved using surrogate endpoints. As it is impossible to control for every medication intake/application, patients need to be monitored by more or less crude subjective measurements as a generally accepted “gold standard” does not exist for measuring adherence and persistence. Following the definition of ISPOR (International Society for Pharmacoeconomics and Outcome Research), adherence is “the degree or extent of conformity to the recommendations about day-to-day treatment by the provider with respect to the timing, dosage, and frequency” [19]. Another way to measure adherence is to estimate persistence, which is defined as the duration of time from initiation to discontinuation of therapy (8;19). A patient is optimally adherent if no doses are missed, no extra doses are taken and no doses are taken in the wrong quantity or at the wrong time. A patient has optimal persistence if they take a medication as long as it is prescribed [20].

### Definition of adherence

Adherence was defined as the percentage of a prescribed dose actually taken within a certain time frame. For this study the adherence after 12 and 24 months after therapy initiation was measured and we differentiated between self-reported adherence and prescription refill counts. Prescription refill counts were used to calculate the Medication Possession Ratio (MPR), which indicated the recommended prescriptions to actual prescriptions quotient. A patient was classified as adherent if self-reported adherence and an MPR of 80% or more was achieved. The limit of 80% was chosen based upon current literature [13,15,21-23]

### Definition of persistence

Persistence was defined as the duration (in months) of therapy from initiation to discontinuation. Discontinuation was defined as no medication (refills/in possession) for at least 60 days or a discontinuation registered in the patient file for whatever reason (e.g. due to side effects).

### Measuring adherence and persistence

This study aimed to investigate the everyday-life setting and only methods that did not stress or influence the patients were used.

To ensure these criteria, we combined two strategies:

1) Self-reported adherence using a specifically designed and standardized questionnaire in combination with an interview [17]. The questionnaire included 10 items concerning aromatase inhibitor tolerance, side effects, adherence and persistence. Furthermore, we added specifically designed questions that addressed side effects, patient’s attitude towards breast cancer, their specific treatment, their knowledge about breast cancer and quality of life.

2) To add more objective criteria, we assessed prescription refills and calculated the medication possession ratio (MPR). Prescribed tablets were evaluated from hospital charts and prescription refill details (i.e. number of tablets prescribed, date of prescription, extra samples donated, etc.) from all physicians involved in the treatment of the patient (e.g. GP, Gynaecologist, Oncologist). To ensure the completeness of the data, the patient, hospital, GP, Gynaecologist and Oncologist were asked to provide information for any physician known to be involved in the prescribing process.

For the primary endpoint, these two measurements were combined and patients classified as adherent if both self-reporting and prescription refills indicated an adherence above 80%. All interviews and the adherence classification were performed in a blinded manner, with interviewers and analysts not being informed about the randomization results.

### Design

A single-centre, three-armed, randomized and partially-blinded parallel group study was designed with the primary analysis at 12 and a secondary analysis planned for 24 months (not evaluated in this paper). Patients diagnosed with hormone receptor positive primary breast cancer that were recommended adjuvant treatment with an aromatase inhibitor were recruited. Diagnosis and treatment were initiated (independent of the study) according to German breast cancer guidelines by the local the interdisciplinary breast cancer tumour board which is part of the local comprehensive cancer centre. Study participation was offered to all patients that met the inclusion criteria.

### Intervention

**Figure 1:**
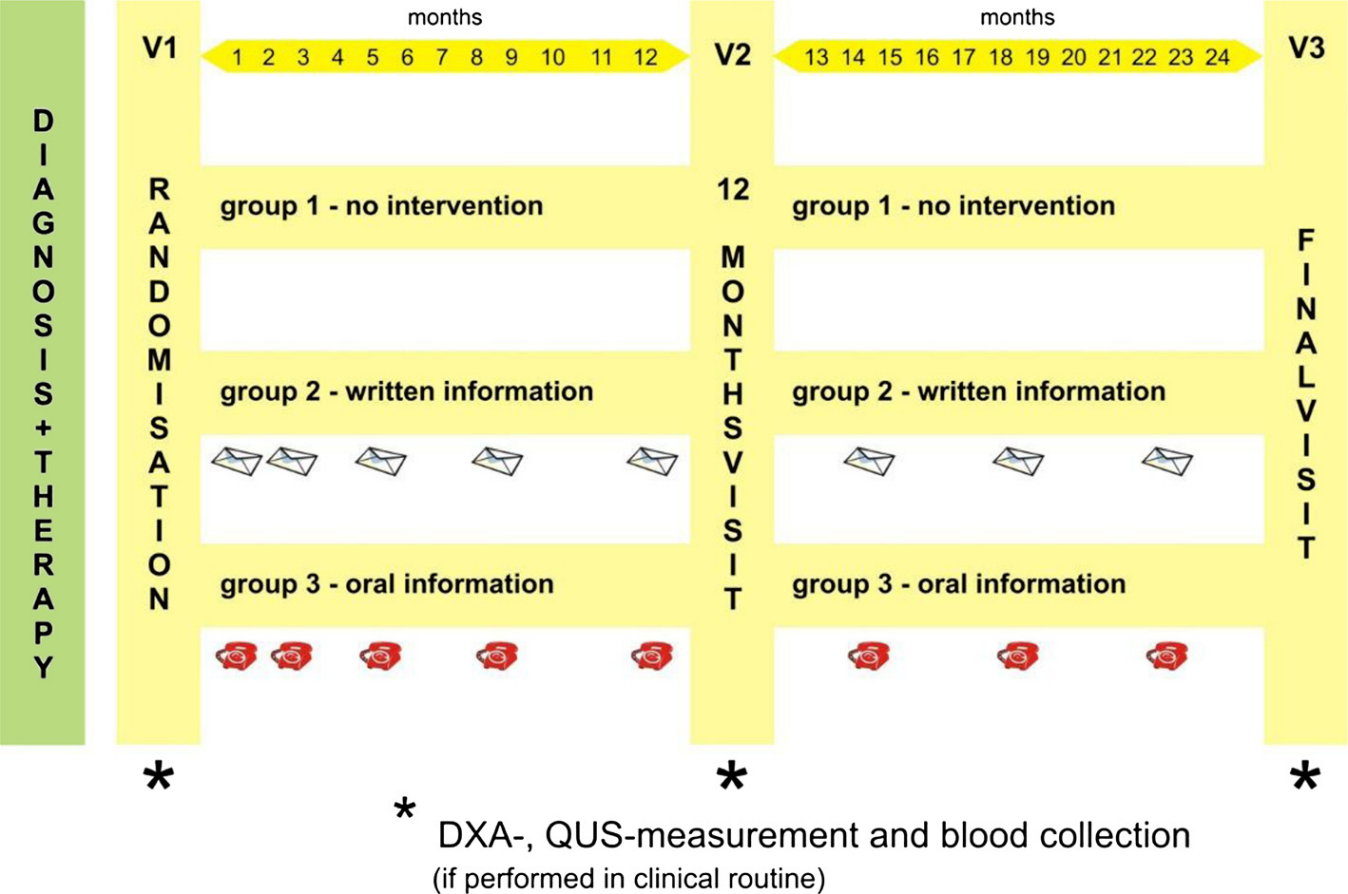
Study scheme.

The intervention aimed to support patients in staying on treatment by reminding, informing and motivating them. Following the psychological principals of learning theory, and using variable, intermittent re-enforcement, the interventions were placed at doubling intervals during the first year. This approach was designed to achieve maximum effect with reasonable practicability. Interventions were planned for week 1, 2, 10, 20 and 33 in the first year (after start of therapy) and months 15, 18 and 21 in the second year (Figure 1).

Group 1 (Control group) – No intervention only standard information provided.

Patients received baseline information in the hospital and the 12 and 24 month interviews (visits).

Group 2 (Letter group) – Patients received a personalized motivational reminder letter, informative content in combination with a breast cancer information leaflet at 1, 2, 10, 20 and 33 weeks and at month 15, 18 and 21.

Patients were addressed personally, reminded of the importance and impact of their disease, as well as the effects and possible side-effects of aromatase inhibitor (AI) treatment. Each letter included contact information and phone number for the study nurse available to answer questions as well as information for when the doctor should be contacted. The leaflet contained different breast cancer related topics such as “sport and cancer”, “nutrition and cancer”, etc.

Group 3 (Telephone group) – Patients were contacted by a study nurse at week 1, 2, 10, 20 and 33 and month 15, 18 and 21 via telephone. Employing a semi-structured interview technique, patients were reminded, informed and motivated during the phone call. The call aimed to provide individualized information, feedback to questions and problems with medication or provide contact with the treating oncologist if needed. Strategies were discussed that ensured the regular intake of the tablets (“what will you do to ensure you don’t forget to take a tablet?”, “Where will you keep the tablets?”, etc.).

### Visits

Patients were randomized to each group and baseline data recorded during the hospital stay for primary treatment. Specifically designed questionnaires were sent to each patient at 12 and 24 months in addition to a telephone interview. Additionally other adherence related parameters and clinical questions were captured.

### Study population

Between April 2006 and December 2008, all patients receiving aromatase inhibitor therapy as an adjuvant treatment for primary breast cancer, were screened for the study.

### Inclusion criteria

•Female

•Primary breast cancer

•Aromatase inhibitor therapy following German breast cancer guidelines [24].

•Informed consent

•Patient capable of using oral medication under their own initiative following prescribing information.

### Exclusion criteria

•Continuously hospitalized, residing in a nursing home or receiving support via an ambulatory home care service (or similar service).

•Suffering from any form of dementia or similar disease interfering with memory.

•Other disease, mental or physical disorder that, in the opinion of the study coordinator, would have interfered with participation in the study.

•Known medical, drug or alcohol abuse.

### Primary and secondary analytic end points and planned analysis

The primary endpoint for the analysis was the proportion of patients that could be classified “adherent” after 12 months according to pre-defined criteria. Adherence was further analysed by self-reported adherence and medication possession ratio. Secondary analysis evaluated persistence and future analyses are planned to analyse possible factors influencing adherence and persistence, reasons for non-adherence and discontinuation, influence of the interventions on other factors as knowledge of the disease and quality of life.

### Statistical analysis

With a group size of 60 per arm (including a drop-out rate of ~10%), the study was designed to detect a minimum interventional effect of 30% with α = 5%, 0.8 power and two-way significance,

All analyses were performed using IBM-SPSS Version 17.0.

Descriptive analyses of the baseline characteristics, as well as testing normal distribution, were performed using the Kolmogorov-Smirnov-Test. Hypothesis testing was performed using Kruskal-Wallis, Chi-squared test or T-test/U-test (Mann–Whitney) where applicable. Wilcoxon-testing was applied for time-dependent tests and Kaplan-Meier-Survival Analysis and Log Rank tests were used to calculate persistence.

### Ethics

The study was conducted in accordance to the guidelines and with approval of the local ethics committee of the Philipps-University of Marburg.

## Results

**Figure 2:**
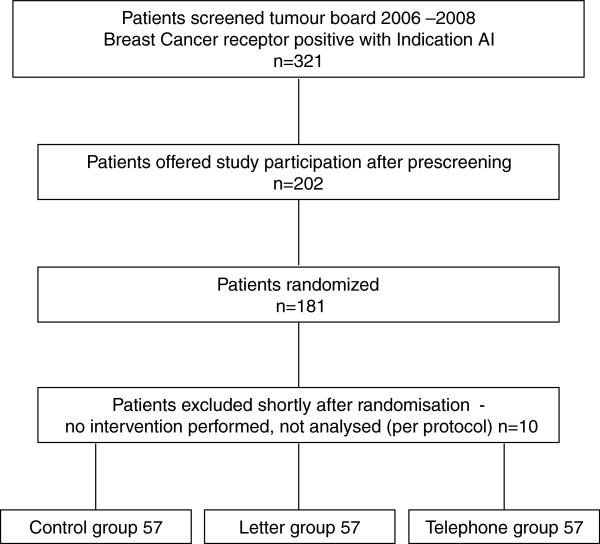
Consort diagram (12 month evaluation).

Patients (181) were recruited and randomized between April 3rd, 2006 and December 18th, 2008 (Figure 2) with a mean age at recruitment of 63.3 (SD 8.9). No significant differences were found in baseline characteristics with regards to age, BMI, tumour characteristics (TNM-Classification, grading, receptor-status), primary and adjuvant therapy, concomitant diseases, number of concomitant medications, profession and others (Table 1). Of these, 10 patients were excluded within four weeks of randomisation as they no longer met inclusion criteria (e.g. withdrew consent; 5 without providing a reason, 2 due to other serious disease, 2 due to starting an externally controlled treatment - home care service, 1 restarted menstruation just after randomisation and was switched to tamoxifen). No further follow-up was performed and they were counted as drop-outs.

**Table 1:** Baseline characteristics

	**Controls**		**Phone group**		**Letter group**		**Total**	
	**n**	**Mean/SD**	**n**	**Mean/SD**	**n**	**Mean/SD**	**n**	**Mean/SD**
Age (years)	57	62,5/8,5	57	64,1/9,8	57	63,2/8,1	171	63,3/8,8
Number Children	57	1,8/1,2	57	1,9/1,1	57	1,91/1,4	171	1,8/1,3
Number concomitant disease	56	5,1/3,2	54	5,4/3,5	55	5,2/3,1	165	5,2/3,2
Number concomitant medications	57	2,5/3,1	54	2,6/2,8	54	2,4/2,4	169	2,5/2,8
Tumour data								
Histology	n	%	n	%	n	%	n	%
Invasiv	56	98,2	58	100	59	100	173	99,4
Microinvasiv	1	1,8	0	0	0	0	1	0,6
Total	57	100	58	100	59	100	174	100
TNM	n	%	n	%	n	%	n	%
T1	33	57,9	37	62,7	37	63,8	107	61,5
T2	17	29,8	17	28,8	20	34,5	54	31
T3	6	10,5	3	5,1	1	1,7	10	5,7
T4	1	1,8	2	3,4	0	0	3	1,7
total	57	100	59	100	58	100	174	100
N0	27	47,4	32	54,2	26	44,8	85	48,9
N1	19	33,3	18	30,5	21	36,2	58	33,3
N2	8	14	5	8,5	6	10,3	19	10,9
N3	3	5,3	4	6,8	4	6,9	11	6,3
NX	0	0	0	0	1	1,7	1	0,6
Total	57	100	59	100	58	100	174	100
M0	56	98,2	57	96,6	57	98,3	170	97,7
M1	1	1,8	2	3,4	1	1,7	4	2,3
total	57	100	59	100	58	100	174	100
Treatment, surgery	n	%	n	%	n	%	n	%
Breast conserving therapy	42	73,7	37	62,7	42	72,4	121	69,5
Mastectomy	14	24,6	22	37,3	16	27,6	52	29,9
Total	57	100	59	100	58	100	174	100
Axillary node dissection (incl. SLN)	56	98,2	54	98,2	53	100	163	98,8
Radiotherapy	n	%	n	%	n	%	n	%
Negativ	2	3,5	6	10,5	4	7	12	7
Positiv	55	96,5	51	89,5	53	93	159	93
Total	57	100	57	100	57	100	171	100
Chemotherapy	n	%	n	%	n	%	n	%
Any	16	28,1	11	19,3	23	40,1	50	29,5
None	41	71,9	46	80,7	33	58,9	120	70,6
Total	57	100	57	100	56	100	170	100

**Figure 3:**
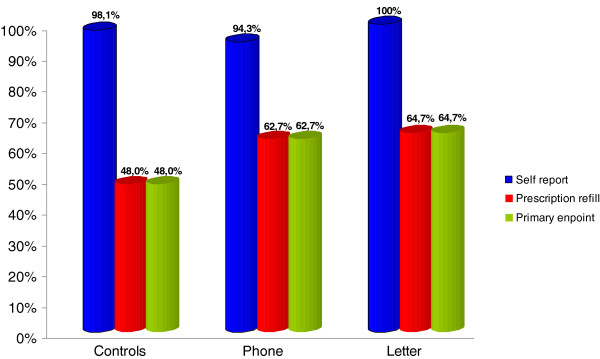
Rate of adherent patients: self report vs prescription refill vs prim endpoint (combined).

The primary group endpoint revealed 48.0% (CI 35–62) of patients in the control group, 64.7% (CI 51–77) in the letter group and 62.7% (CI 49–75) in the phone group were classified as adherent after 12 months (Figure 3). The primary endpoint was comprised of self-reported adherence and prescription refill counts. For self reported adherence alone, the analysis revealed 98.1% (CI 92–100), 94.3% (CI 87–99) and 100% (CI 96–100) adherent patients for controls, letter and phone group, respectively (Figure 3). Prescription refills (80% cut-off) were achieved in 48% (CI 35–62), 64.7% (CI 51–76) and 62.7% (CI 48–74) in the control group, letter group and phone group, respectively. The differences between the groups did not reach statistical significance (Figure 3). A logistic regression analysis did not show any significant influence on adherence for t-stage, nodal status, chemo- and radiotherapy and intervention group when entered as covariants in the model. To further analyse the trend seen for the primary endpoint and to enhance the power of the analysis, a pooled analysis with a one-way hypothesis for both interventions versus control was performed. This post hoc testing indicated a significant difference between the groups (p = 0.039).

**Figure 4:**
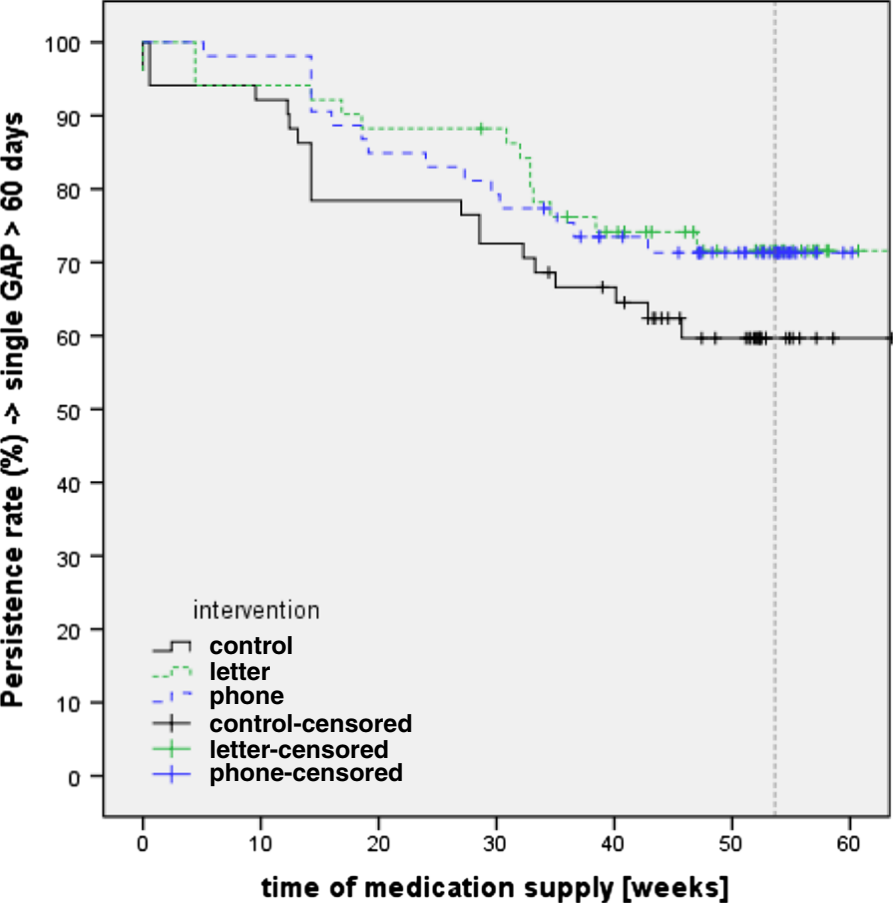
Persistence rate.

Persistence was evaluated using Kaplan-Meyer Survival analysis and results are shown in Figure 4. The Log Rank (Mantel-Haenszel) Test showed no significance between intervention groups (χ^2^ = 2.39; p = 0.305; df = 2). Discontinuation was only based on medication refill, in no case patient file information lead to further discontinuation events. Mean medication possession ratio and persistence is provided in Table 2. Mean Medication Possession Ratio (MPR) and persistence were not normally distributed by the Kolmogorov-Smirnov-Test. Overall significance between groups was tested using the Kruskal-Wallis-Test for MPR (χ^2^ = 3.83; p = 0.147) and persistence (χ^2^ = 5.01; p = 0.082). A U-Test (Mann–Whitney) for mean MPR did not reach statistical significance when comparing control and letter groups (Z = 1.85; p = 0.064), no significance between control and phone groups (Z = 1.48; p = 0.145) and between intervention groups (Z = 0.44; p = 0.660).

**Table 2:** Mean MPR and persistence

	**Control**		**Letter**		**Phone**		**Total**	
		**N = 51**		**N = 51**		**N = 53**		**N = 155**	
	**Mean**	**SD**	**Mean**	**SD**	**Mean**	**SD**	**Mean**	**SD**	
MPR (%)	70.4	31.8	79.7	27.9	78.4	26.5	76.2	28.9
Persistence (weeks)	38.6	17.5	44.7	16.8	42.8	14.8	42.1	16.5

When mean persistence was analysed, the difference between control and letter groups (Z = 4.50; p = 0.034) was significant, while no significant differences were found between control and phone groups (Z = 1.55; p = 0.121) or between interventions (Z = 0.797; p = 0.426).

Self reported global medication tolerance at the 12 month visit was bad or very bad in 23,6% of all patients (Table 3). Grouping was performed according to primary endpoint. When asked for reasons for interrupting treatment, 7.5% of all patients admitted forgetting to take the medication while 68% of those with treatment gaps did so due to side effects. No significant differences were seen between groups.

**Table 3:** Tolerance

	**Non-compliant**		**Compliant**		**Total**	
	**N**	**%**	**N**	**%**	**N**	**%**
Very bad	9	**14.3**	9	**10.1**	18	11.8
Bad	10	**15.9**	8	**9.0**	18	11.8
Indifferent	13	**20.6**	29	**32.6**	42	27.6
Good	21	**33.3**	27	**30.3**	48	31.6
Very good	10	**15.9**	16	**18.0**	26	17.1
Total	63	100.0	89	100.0	152	100.0

## Discussion

The well-established benefits of an adjuvant endocrine treatment with aromatase inhibitors can only be achieved if patients adhere to prescribed medications. A lack in adherence may lead to reduced clinical outcomes, unnecessary change of treatment, increased side effects or even higher hospitalisation rates due to treatment failure [20]. Despite the fact that women on adjuvant endocrine treatment for breast cancer are generally expected to be highly adherent as they are facing a serious life threatening disease, the treatment is effective, easy to use and generally well-tolerated, recent studies underline a clinically relevant decrease in adherence to tamoxifen and aromatase inhibitor therapy (including a reduction in cancer outcome) as soon as 12 months [13,14,25]. This indicated that patient statements and the doctors’ perception with regards to adherence might not always reflect reality.

Waterhouse et al. compared self-reported adherence, classical pill count and the use of a micro electronic monitoring device (MEMS) in tamoxifen patients and found a significantly higher level of adherence was recorded by patient self–reporting when compared to MEMS (16.7% vs. 29% non-adherence at 3 months) [16]. Other studies evaluating adherence to tamoxifen, using self-reported evaluation or database claim methods, found adherence rates ranging from 65% to 85% for different periods of follow-up [13,26-29]. Barron et al. demonstrated using prescription refill counts that by the end of follow-up at 3.5 years, the cumulative non-persistence rate had increased to 35.2% [30]. In an analysis by Partridge et al. within the used claim databases, the mean adherence to anastrozole significantly decreased from 78%-86% during the first year to 62%-79% during the third year [15]. The authors themselves, using the same methodology described for the COMPAS study comparing adherence and persistence to tamoxifen and anastrozole in the clinical practice, reported reduced adherence rates for the adjuvant treatment of breast cancer [17]. This served to illustrate the need for research to study and implement strategies helping patients to stay on medications and adhere to their treatment. However, improving adherence to aromatase inhibitor medication/treatment is a complex and challenging issue that has not been sufficiently studied [21].

We developed the COMPAS study in order to evaluate the clinical efficacy of two simple/viable interventions for improving adherence to adjuvant treatment with an aromatase inhibitor in breast cancer patients. These interventions provided a multifaceted approach to improved knowledge of the disease, treatment advantages and disadvantages in addition to explanations and solutions for the patient. The semi-structured interview style of the phone call from health professionals is designed to enhance the effects by using elements of motivational interviewing [31-33].

To measure adherence/persistence with a combination of self-reported information and prescription refill counts was a practical and fairly objective way to assess adherence in a real-life setting. The evaluation of the baseline characteristics showed a well-balanced randomization and a representative sample structure of the investigated patients while the baseline demographic characteristics of our real-life sample were comparable to study populations in pivotal aromatase inhibitor trials with regard to age, tumour characteristics and primary therapy [1-3]. They also show that the German guidelines for treatment initiation had been followed as suggested [24].

Evaluation of the primary endpoint revealed marked differences between the groups, even though the differences did not reach statistical significance. The authors are convinced there was a clinically significant effect for the interventions as demonstrated by the numerical increase for both interventions. Additionally, after pooling the intervention groups, a significant difference could be found in mean persistence. This cannot be taken as a final proof of concept, but should encourage the development of further interventions and studies containing a greater number of patients.

Kaplan-Meyer analysis for persistence showed that after one year a marked number of patients had discontinued treatment. There was a slight but statistically insignificant improvement due to the interventions. The reasons for non-adherence and non-persistence were diverse and the authors believe that only interventions that address multiple issues in the patients everyday life would lead to significant improvements.

Several publications have linked tolerance to adherence [13]. The COMPAS study showed a positive side-effect profile, with 49.7% of patients reporting good or very good tolerance to their aromatase inhibitor

Non-adherent patients had slightly lower tolerance, but the difference was not statistically significant. There were no differences in the rate of patients that stopped treatment due to tolerance issues. Furthermore no other baseline parameters showed significant associations to adherence outcome. From the patients view, remembering to take the medication did not seem to be an issue as only 7.5% reported “forgetting” to take the tablets. Interventions that could enhance adherence would need to specifically address individual problems that occurred over time and due to individual habits, side effect(s), coping strategies, health belief and others.

## Conclusions

COMPAS was designed to use personalized, multifaceted approaches to support numerous patients with a viable cost effective approach. Even so, the effects on adherence and persistence were not as significant as expected. Assisting patients with taking their medications should improve therapeutic outcome and efficiency, which would be of upmost importance to the individual, therefore further scientific effort is needed to reach those aims.

## Competing interests

Professor Hadji has received honoraria from Astra Zeneca, Pfizer and Novartis as a consultant/advisor.

The other authors have nothing to disclose.

## Authors’ contributions

VZ & PH conceived of the study, participated in its design and coordination, drafted the manuscript and made substantial contributions to analysis and interpretation of data. IK participated in the design of the study, made substantial contributions to acquisition of data and helped to draft the manuscript. DK & AS made substantial contributions to acquisition of data and helped to draft the manuscript. OH participated in the design of the study and performed the statistical analyses. All authors read and approved the final manuscript.

## Pre-publication history

The pre-publication history for this paper can be accessed here:

http://www.biomedcentral.com/1471-2407/13/407/prepub
